# Prenatal Immune and Endocrine Modulators of Offspring's Brain Development and Cognitive Functions Later in Life

**DOI:** 10.3389/fimmu.2018.02186

**Published:** 2018-09-26

**Authors:** Steven Schepanski, Claudia Buss, Ileana L. Hanganu-Opatz, Petra C. Arck

**Affiliations:** ^1^Laboratory of Experimental Feto-Maternal Medicine, Department of Obstetrics and Fetal Medicine, University Medical Center Hamburg-Eppendorf, Hamburg, Germany; ^2^Developmental Neurophysiology, Institute of Neuroanatomy, University Medical Center Hamburg-Eppendorf, Hamburg, Germany; ^3^Institute of Medical Psychology, Berlin Institute of Health, Charité-Universitätsmedizin Berlin, Corporate Member of Freie Universität Berlin, Humboldt-Universität zu Berlin, Berlin, Germany; ^4^Development, Health, and Disease Research Program, University of California, Irvine, Orange, CA, United States

**Keywords:** pregnancy, fetal brain development, prenatal infection, maternal distress, maternal microchimeric cells, cytokines, glucocorticoids (GC), epigenetic aberrations

## Abstract

Milestones of brain development in mammals are completed before birth, which provide the prerequisite for cognitive and intellectual performances of the offspring. Prenatal challenges, such as maternal stress experience or infections, have been linked to impaired cognitive development, poor intellectual performances as well as neurodevelopmental and psychiatric disorders in the offspring later in life. Fetal microglial cells may be the target of such challenges and could be functionally modified by maternal markers. Maternal markers can cross the placenta and reach the fetus, a phenomenon commonly referred to as “vertical transfer.” These maternal markers include hormones, such as glucocorticoids, and also maternal immune cells and cytokines, all of which can be altered in response to prenatal challenges. Whilst it is difficult to discriminate between the maternal or fetal origin of glucocorticoids and cytokines in the offspring, immune cells of maternal origin—although low in frequency—can be clearly set apart from offspring's cells in the fetal and adult brain. To date, insights into the functional role of these cells are limited, but it is emergingly recognized that these maternal microchimeric cells may affect fetal brain development, as well as post-natal cognitive performances and behavior. Moreover, the inheritance of vertically transferred cells across generations has been proposed, yielding to the presence of a microchiome in individuals. Hence, it will be one of the scientific challenges in the field of neuroimmunology to identify the functional role of maternal microchimeric cells as well as the brain microchiome. Maternal microchimeric cells, along with hormones and cytokines, may induce epigenetic changes in the fetal brain. Recent data underpin that brain development in response to prenatal stress challenges can be altered across several generations, independent of a genetic predisposition, supporting an epigenetic inheritance. We here discuss how fetal brain development and offspring's cognitive functions later in life is modulated in the turnstile of prenatal challenges by introducing novel and recently emerging pathway, involving maternal hormones and immune markers.

## Introduction

The vertical transmission of maternal immune and endocrine markers is increasingly recognized to modulate fetal neurodevelopment and future mental health of the offspring. Emerging evidence arising from observational studies in humans reveals that prenatal environmental challenges such as maternal distress perception and infections are associated with an impaired fetal neurodevelopment and increased risk for neurological or psychiatric disorders later in life (1–6). Insights into the underlying mechanisms and pathogenesis of prenatally programmed poor mental health are increasingly emerging. It is well known that neurodevelopment results from the interaction of genetic, epigenetic and environmental factors, through which proliferation, migration of neural progenitor cells and establishment of neuronal circuits are modulated. Disruptions of these neurodevelopmental pathways may subsequently affect future brain function, as reflected by cognitive and intellectual impairment and increased the risk for neurodevelopmental and psychiatric disorders later in life (7).

Here, we compile the currently available evidence arising from observational studies supporting the concept of a developmental origin of brain disorders. We further outline cornerstones of brain development in mice and humans and discuss the effect of prenatal challenges, primarily maternal distress and infections, on maternal immune-endocrine adaptation to pregnancy. Lastly, we introduce novel concepts on how an altered maternal immune-endocrine adaptation to pregnancy can impact on offspring's brain development and subsequent mental health.

### Developmental origin of neurological dysfunctions and psychiatric disorders

Pregnancy is characterized by significant adaptational processes of the maternal immune and endocrine system in order to ensure its progression until term, which is required for adequate fetal development. These adaptational processes are highly responsive and vulnerable to challenges, such as high maternal stress perception or maternal infections. In this context, chronic stress states (e.g., depression or anxiety) affect approx. 10–15% of pregnant women worldwide (8). Moreover, negative life events may pose a significant threat to maternal wellbeing during pregnancy. Table 1 provides a comprehensive overview of published evidence largely arising from observational studies that reveal a significant association between various types of maternal distress perception to which the mother was exposed to at specific gestational periods and the risk for psychiatric disorders in the offspring later in life, i.e., during childhood or adolescence. Disorders observed in the offspring include autism spectrum disorder (ASD) (9), depressive symptoms (10–12), anxiety, borderline personality disorder, eating disorders (23) and attention-deficit/hyperactivity disorder (ADHD) (3, 14–20, 22, 45). Interestingly, whilst a sex-specific risk is well known for psychiatric disorders, only very few studies paid attention whether prenatal stress perception skews the risk for such disorders in a sex-specific way. One study describes a sex-bias for ADHD upon prenatal stress, mirrored by a higher risk in daughters (20). Moreover, the timing of the prenatal challenge may be pivotal, as the risk for neurodevelopmental diseases appears to be differentially affected by the trimester of exposure (Table 1). In fact, surges of maternal IL-6 levels during the third trimester—which may result from distress or infections—showed a strongest impact on working memory performance in children. These behavioral changes were associated with alterations of brain regions tightly associated with working memory, as identified by functional MRI (46).

**Table 1:** Summary of human studies examining the effect of prenatal maternal distress on offspring's mental health.

**Proxy for prenatal maternal distress**	**Offspring's age at outcome evaluation**	**Offspring's outcome**	**Reference**
**INCIDENCE DURING THE FIRST TRIMESTER**			
Exposure to a natural disaster	Childhood-preadolescence	ASD symptoms	(9)
Questionnaires-based evaluation of stress perception	Adolescence-adulthood	Internalizing and externalizing problems Depressive symptoms	(10–12)
Self-reported stressful events	Birth-adulthood	No risk for psychosis	(13)
**INCIDENCE DURING THE SECOND TRIMESTER**			
Exposure to a natural disaster	Childhood-preadolescence	ASD symptoms	(9)
Questionnaires-based evaluation of depression	Childhood-adolescence	Internalizing and externalizing problems Anxiety symptoms Depressive symptoms Hyperactivity Borderline personality disorder	(3, 14–18)
Self-reported stressful events	Childhood-adolescence	Internalizing problems Depressive symptoms ADHD symptoms No association to total psychiatric problems No risk for psychosis	(13, 19–21)
Questionnaires-based assessment of anxiety	Child-preadolescence	Total psychiatric problems Internalizing and externalizing problems Anxiety symptoms Depressive symptoms Hyperactivity	(22, 14)
Saliva cortisol	Preadolescence	ADHD symptoms especially in boys	(21)
**INCIDENCE DURING THE THIRD TRIMESTER**			
Exposure to a natural disaster	Childhood-preadolescence	ASD symptoms Eating disorder symptoms	(9, 23)
Questionnaires-based evaluation of depression	Preadolescence-adolescence	Externalizing problems Hyperactivity	(18, 24)
Self-reported stressful events	Birth–adulthood	ADHD symptoms No association to total psychiatric problems No risk for psychosis	(13, 20, 21)
Questionnaires-based assessment of anxiety	Childhood-adolescence	Internalizing problems Depressive symptoms Hyperactivity	(3, 14, 15)
Saliva cortisol	Preadolescence	ADHD symptoms especially in girls	(21)
Physician-based diagnoses of depression	Childhood-adulthood	Depressive symptoms ADHD symptoms	(6, 25)
ICD-based diagnoses of anxiety	Childhood	ADHD symptoms	(6)
**INCIDENCE DURING PREGNANCY WITHOUT FURTHER TRIMESTER SPECIFICATION**			
Self-reported stressful events	Childhood-adulthood	Eating disorders ASD symptoms Schizophrenia in male offspring	(26–28)

Besides high stress perception, maternal infection during pregnancy can interfere with fetal neurodevelopment and increase the risk for neurological dysfunctions and psychiatric disorders in the offspring (Table 2). Here, most of the studies focus on the distinct pathogens that have led to maternal infection during pregnancy (1, 35, 37, 42, 43). For example, maternal infection with influenza A or B virus has been associated with an increased risk for developing schizophrenia, although findings between studies are highly ambiguous and hence, hotly debated. Some studies report that influenza infection during the first trimester may trigger the risk for schizophrenia, whilst such effect could not be confirmed in studies focusing on infection at mid to late pregnancy (29–31, 47). The latter includes observations arising from Scandinavian registry analyses, where the query for prenatal influenza infection was solidly based on International Classification of Diseases (ICD)-coded diagnoses (48, 49). A recent meta-analysis confirms that evidence is insufficient to support gestational influenza as a risk factor for schizophrenia and bipolar disorder in the offspring (50). Besides these viral infections, also bacterial infections during pregnancy have been linked to an increased risk for schizophrenia in the offspring in adulthood (36, 44). For instance, 13% of all children surviving the maternal listeria monocytogenes infection were suffering from meningitis in early childhood (38) and this in return is significantly associated with developing schizophrenia and psychotic episodes later in life (39).

**Table 2:** Overview of studies examining the effects prenatal infection and related maternal immune activation on offspring's mental health in human.

**Gestational time point of infection**	**Proxy used to identify prenatal infection**	**Offspring's age at evaluation**	**Offspring's outcome**	**Reference**
**VIRAL INFECTIONS DURING PREGNANCY**				
Each trimester	Maternal antibodies against Influenza virus A and B	Adulthood	Increased risk for schizophrenia	(29–31)
End of pregnancy	Maternal antibodies against cytomegalovirus, rubella virus, human parvovirus B19, herpes simplex virus 1 and 2	Lifetime	Increased risk for schizophrenia Increased risk for psychosis	(32, 33)
Newborn's blood (5–7 days old)	Maternal antibodies against herpes simplex virus 2	Lifetime	Increased risk for schizophrenia	(34)
Pregnancy	Retrospective estimation infection	2–5 years	Maternal fever, but not influenza, increases the risk for schizophrenia	(35)
**BACTERIAL INFECTIONS DURING PREGNANCY**				
End of pregnancy	Maternal antibodies against chlamydia trachomatis	Lifetime	Increase risk for schizophrenia	(32)
Pregnancy	Retrospective estimation	32–34 and 45–47 years	Increased risk for schizophrenia	(36)
Pregnancy	Maternal C-reactive protein	ASD diagnosis	Increased risk for ASD	(37)
Pregnancy	ICD-based registry queries	–	Increased risk for schizophrenia and psychotic episodes via meningitis in childhood	(38, 39)
**PARASITIC INFECTIONS DURING PREGNANCY**				
Each trimester	Maternal antibodies against toxoplasma gondii	24–30 years	Increased risk for schizophrenia	(30, 40)
End of pregnancy	Maternal antibodies against toxoplasma gondii	Lifetime	Increased risk for schizophrenia	(41)
**NON-CLASSIFIED PRENATAL INFECTIONS**				
Pregnancy	ICD-based registry queries	Lifetime	Increased risk for ASD Increased severity of ASD symptoms compared to non-infected ASD offspring	(1, 42, 43)
Pregnancy	ICD-based registry queries	Lifetime	Maternal infection before and after pregnancy increased the risk for schizophrenia	(44)

### Milestones of brain development in mice and humans

Given that some of the observational studies summarized above highlight that the time point at which challenges occur prenatally may be crucial to trigger changes in fetal neurodevelopment, we here provide a brief summary of key aspects of fetal brain development that commence or are completed at distinct periods of gestation. We also include the brain development of mice, as mouse models have become pivotal in understanding how prenatal challenges affect neurodevelopment and brain functions later in life, as outlined in the subsequent paragraphs.

**Figure 1:**
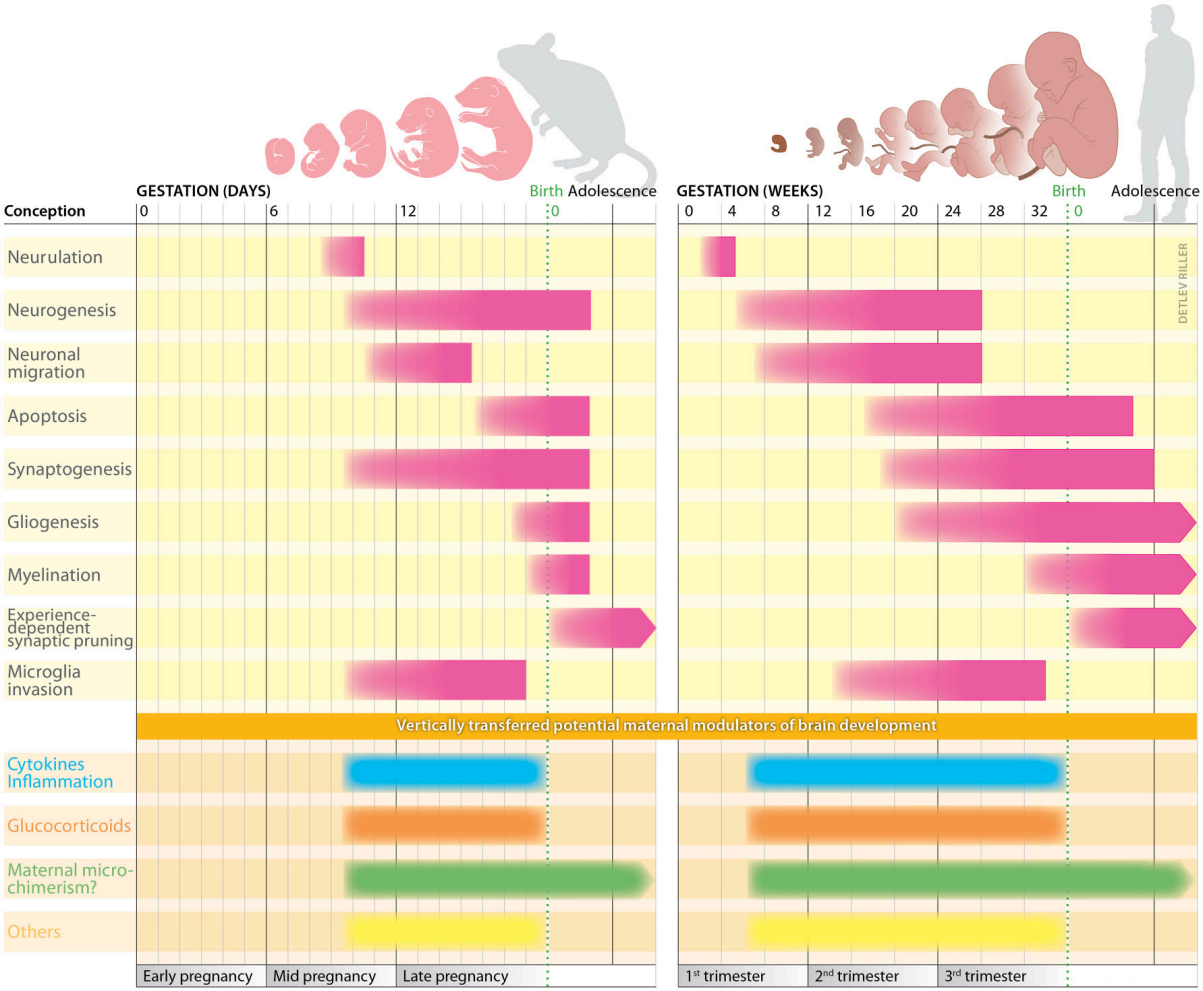
Milestones of brain development in mice and humans. In **both species**, brain development commences with neurulation, a process creating the neural tube. This provides the prerequisite for the subsequent production of neuron from neural stem cells, a process defined as neurogenesis. Early during human development, at gestation week 4, the anterior part of the neural tube begins to form into distinct regions. The forebrain, midbrain and hindbrain are defined as the anterior part; the spinal cord is located at the posterior part. Two weeks later, the neural tube can be clearly divided into the brain regions that are present at birth. Some of the previously produced neurons now start to migrate to distinct brain regions, a process that continues until approx. week 26. Earlier by week 11, the cerebrum has developed—more rapidly than other structures—and largely covers the entire brain, except cerebellum and medulla oblongata. Due to its progressive development within the cranium, the cerebrum is forced to convolve itself resulting in gyri and sulci (51). During the second trimester of pregnancy, several processes start to define brain connectivity. These include synaptogenesis, gliogenesis, and apoptosis. Simultaneously, microglial cells invasion begins. By mid-third trimester, the fine-tuning of neuronal connectivity starts with proliferation of myelin sheaths throughout the neurons of the central nervous system (52). Shortly after birth, the previously established neuronal connections are reduced based on neuronal activity, meaning a reduction of neuronal connections to the ones often used. Since murine gestation is much shorter compared to human pregnancy, some developmental steps continue to proceed after birth. In mice, neural development begins during mid-pregnancy, followed by neurulation and formation of the neural tube (53). Subsequently, production of neurons, their migration and the formation of synapses occur almost simultaneously. Also, yolk sac-derived microglial cells invade the fetus starting on day 9 of pregnancy (54). The developmental milestones underlying brain development in mice and humans are highly susceptible to challenges and can be modulated by maternal markers vertically transferred during pregnancy. Hereby, the time point and intensity of the challenges clearly determine the impact and damage they may cause.

In humans and mice, brain development shows similar developmental processes (Figure 1). Noteworthy, sex-specific differences occur as a result of a faster cerebral maturation in girls (55), which leaves boys at higher risk for challenges-induced disruptions due to the greater window of vulnerability. Some developmental steps continue after birth in mice, which are already completed at birth in humans. This reduces the time window in mice during which vertically transferred maternal biological mediators can impact on offspring's brain development, a confounder which should be considered when discussing the biological relevance of findings on prenatal challenges in mice. Hence, when evaluating the impact of maternal markers on fetal development in mice, research endeavor should focus on milestones that are completed prior to birth, such as neurulation, neuronal migration and microglia invasion, as well as synaptogenesis and neurogenesis, the latter being largely completed at birth.

### Effect of prenatal challenges on maternal immune-endocrine adaptation to pregnancy

In mice and humans, healthy brain development is crucially dependent on an endocrine and immunological homeostasis of the mother-fetus dyad. Here, the balance between pro- and anti-inflammatory cytokines is crucial, as neurogenesis, migration, differentiation and apoptosis are well known to be responsive to cytokine challenges (Table 3). Besides cytokines, chemokines are also important in modulating neurodevelopment and the risk for psychiatric diseases. Since these interactions have been addressed in recent reviews (80–82), we refrain from including them here. Similarly, glucocorticoids, which are initially largely maternally derived during gestation in the fetus, can interfere with fetal brain development in mice and humans. Moreover, additional factors such as maternal immune cells can affect fetal brain development. Prenatal stimuli and challenges, including maternal stress perception and infection, have been show to interfere with the endocrine and immunological hemostasis in the mother. This includes the activation of the sympathetic nervous system and the hypothalamus-pituitary-adrenal (HPA) axis, subsequently leading to an excess secretion and availability of free, biologically active glucocorticoids (83, 84). Pregnancy itself is considered to be a state of “hypercortisolism,” which is an essential requirement to meet the maternal demand for increased metabolism and energy generation. The fetus is also critically dependent on the transfer of maternal glucocorticoids, which is controlled for by placental enzymes such as 11ß-Hydroxysteroid-Dehydrogenase (11ß-HSD)-1 and -2 (83, 84). Maternal glucocorticoids ensure structural and functional development of fetal organs, as the fetus is not capable of producing glucocorticoids until late in development. However, elevated glucocorticoid concentrations in the context of maternal stress may negatively impact on fetal brain development (83).

**Table 3:** Key cytokines influencing neural cell development.

**Cytokine**	**Functional outcome**	**Reference**
IL-1α	Supports astrocyte lineage commitment	(56)
	Increases neurogenesis	(57)
	Increases microglia activity	(58)
IL-1β	Inhibition of neurogenesis	(59–61)
	Supports astrocyte lineage commitment	(58, 59)
IL-6	Supports establishment synaptic connectivity	(62, 63)
	Suppresses astrocyte development	(64)
TNF-α	Inhibition of neural progenitor cell proliferation and differentiation (via TNF receptor 1)	(65)
	Supports cell survival (via TNF receptor 2)	(65)
	Neuroprotective functions	(66)
IL-2	Increases differentiation of neural progenitor cells	(67)
	Increases neurogenesis	(68)
IFN-γ	Supports differentiation, migration and neuronal outgrowth (via microglia activation)	(69)
IL-10	Increases neural progenitor cell survival, differentiation and neuronal myelination	(70)
	Supports oligodendroglia progenitor cell survival Increases neural progenitor cell migration	(71) (72)
		
IL-4	Neuroprotection (via microglia activation)	(69)
TGF-β	Excessive concentration leads to declined neurogenesis	(73)
LIF	Promotion of cell growth (via inhibition of differentiation)	(74)
	Supports neural stem cell self-renewal	(75, 76)
IL-17	Inhibition of adult neurogenesis and synaptic function, decreases neural stem cell numbers, proliferation and differentiation	(77–79)

Besides the effect of prenatal challenges on maternal glucocorticoid levels, the maternal immune response may also be skewed toward inflammation in the context of stress in human pregnancy or infection (85, 86). Similarly in mice, maternal stress challenges have been shown to increase levels of pro-inflammatory cytokines in dams (87) and decrease tolerogenic markers such as CD4^+^ regulatory T cells (88). Similarly, prenatal infection e.g., with influenza A virus in mice leads to an increased type 1 response, along with an increased production of pro-inflammatory cytokines, compared to non-infected pregnant mice (89). Equally, the use of “danger signals” such as lipopolysaccharide to induce an inflammatory response in pregnant mice resulted in a collapse of immune tolerance toward the fetus (90).

Maternal cytokines, glucocorticoids as well as maternal immune cells can cross the placenta. Whilst it is difficult to determine if cytokines are maternally derived or produced by the fetus, maternal immune cells can be clearly identified in the offspring. These maternal microchimeric cells can persist in the offspring long after birth (91). Hence, opposed to the vertical transfer of maternal cytokines and glucocorticoids, maternal microchimeric cells may be capable to continuously modulate brain function in the offspring even after birth.

### Impact of altered maternal immune-endocrine adaptation to pregnancy and offspring's brain development and mental health

The vertical transfer of maternal immune and endocrine markers is increasingly recognized to modulate fetal neurodevelopment and future mental health of the offspring. As mentioned above, the impact of cytokines on fetal brain development has been well studied and it is widely accepted that milestones of physiological fetal brain development are modulated by cytokines (Table 3). Hence, exposure to an imbalanced cytokine response during fetal life may disturb fetal brain development, thereby increasing the risk for neurodevelopmental disorders (92).

In mice, fetal exposure to an altered, pro-inflammatory maternal cytokine response can affect brain morphology, mirrored by e.g., an increased pyramidal cell density or reduced neurogenesis (93–96). Similarly, prenatal maternal treatment with the immunostimulant polyinosinic:polycytidylic acid (poly I:C) to mirror some effects of a viral infection has been shown to result in reduced axonal size, myelin thickness and cortical volume of the hippocampus and amygdala in rodent offspring (97, 98). A general maternal immune activation during pregnancy has also been shown to cause presynaptic deficits in hippocampus (99), pro-inflammatory activation in hippocampal microglia (100) or an increase of microglial cell frequencies in the fetal brain (101). Hence, a wealth of studies has shown that maternal immune activation during pregnancy adversely affects fetal brain development on multiple levels. Yet, it is difficult to comprehensively pinpoint distinct pathways, as the studies performed to date have been rather diverse with regard to the species, the gestational time point or the cause of maternal immune activation (102). In humans, a great deal of research focused on the role of maternal levels of interleukin-6 as a proxy for a prenatal inflammatory challenge. Key observations include that maternal IL-6 levels affects offspring's structural and functional connectivity already at the time of birth (103), delaying the development of sensory and cognitive processing (46, 104).

Similar to cytokines, fetal exposure to elevated concentrations of maternal glucocorticoids has been proposed to exert long-lasting, partly sex-specific effects on offspring's brain morphology and function in rodents but also in humans. This includes decreased dendritic morphology and neuronal volume in hippocampi of both sexes in mice, whereas an increased dendritic branching has been detected only in females (105). Another study using mouse models reports an increased spine density, dendritic length and other morphological features in pyramidal neurons of prenatally stressed males, which was decreased in females (106).

In humans, elevated maternal cortisol concentrations in early pregnancy have been associated with larger amygdala volumes, an altered neural connectivity, along with affective symptoms and internalizing problems in girls (107). Interestingly, moderately elevated maternal cortisol levels in late pregnancy could be associated with greater cortical thickness primarily in frontal regions and enhanced cognitive performance in children (108). These findings point to the importance of considering the moderating role of sex and timing of exposure to glucocorticoids during pregnancy.

Since the experimental or study designs differ with regard to stress paradigms/glucocorticoids applications, time point of prenatal interventions or postnatal analyses, species and read-out parameter (109–111), it is not yet possible to comprehensively summarize the outcome of these studies beyond the statement that prenatal stress and related glucocorticoid surges induce sex- and brain area-specific differences in neuronal complexity and neurogenesis. This may subsequently lead to altered cognitive functions later in life.

Microglial cells, which are the resident macrophages of the CNS, have also been extensively studied in response to prenatal stress or prenatal glucocorticoid application. Due to their phagocytic phenotype, microglia have a functional role in remodeling, shaping and pruning of synapses (112). And indeed, prenatal corticosterone application increased the microglial density in the embryonic brain overall and promoted their amoeboid phenotype (113). Interestingly, this result was supported by isolating and culturing P1-2 microglial cells, showing that they are more likely to acquire an amoeboid phenotype (114). At postnatal age, stress increased the total glia cell number in the hippocampus of female but not male offspring (115). One study showed an increased number of microglia cells in the dentate gyrus, suggesting an adverse effect on neurogenesis and simultaneously no changes in the CA1 of the hippocampus (116, 117). The early stress exposure seems to support phagocytic microglia function in the early brain and potentially stimulate their activity throughout early murine lifetime. Their role as a potential mediator between prenatal stress and early neurobiological changes remains vague, especially in other important brain areas such like the PFC.

The barrier between periphery and the brain is an essential interface for communication between both compartments. Even though the blood brain barrier is supposed to be well established during early development, its function in stress and cytokine-related diseases is poorly investigated. However, its permeability is a key component in how maternal markers may influence the offspring's brain development. This topic is excellently described in an comprehensive review elsewhere [see review, (118)] and thus not explained here.

**Figure 2:**
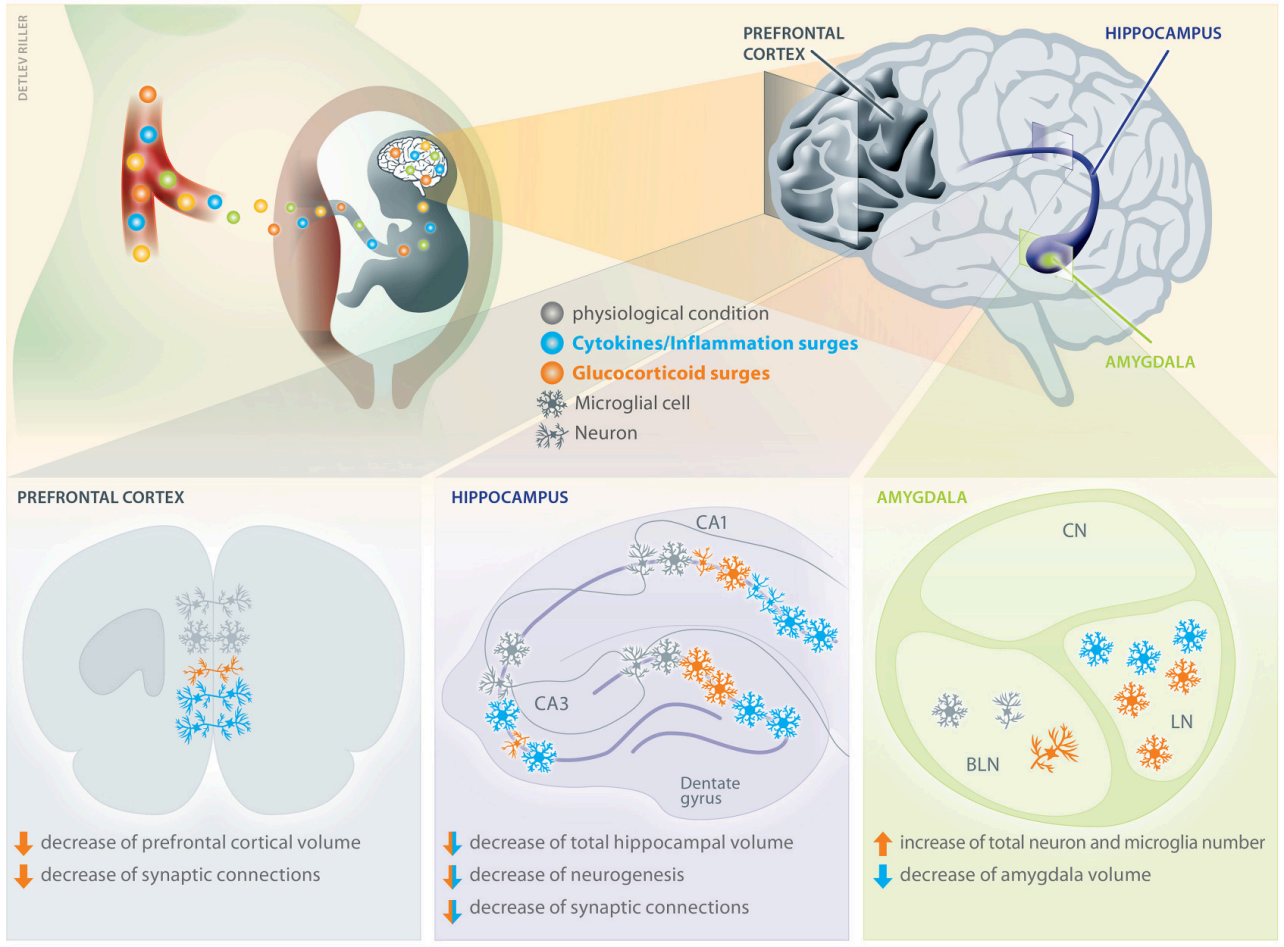
Consequences of cytokine and glucocorticoid surges on distinct areas of the offspring's brain. Maternal cytokines and glucocorticoids can transplacentally cross into the fetus and differentially affect offspring's brain development by interfering with e.g., cell differentiation, axonal growth, and synaptic connectivity. The brain regions depicted here are of pivotal relevance for mental health due to their involvement in cognitive functions. Compared to physiological conditions, prenatal surges of maternal cytokines increase the number of neuronal connections in a subdivision of the prefrontal cortex, whereas glucocorticoids decreases them (105, 119). Additionally, prenatal glucocorticoid exposure decreases prefrontal cortical volume. In the hippocampus, an increase of cytokines is known to increase the number of microglial cells in the corn ammonis area 1 (CA1), whilst simultaneously reducing dendritic arborizations and neuronal complexity (111). Similarly, in the CA3 and dentate gyrus (DG) of the hippocampus, the microglia density increases after cytokine exposure. Also glucocorticoid surges deteriorate the neuronal complexity in CA1 and CA3 (109, 110) and have been shown to increases the number of microglia in DG. Both, cytokines and glucocorticoids can decrease total hippocampal volume, neurogenesis and synaptic connections. Prenatal cytokine surges can also decrease the amygdala volume, whereas glucocorticoids have been shown to increase the number of neurons and microglia. There is no evidence that the central nucleus (CN) of the amygdala is affected, but an increased microglia density after prenatal cytokine and glucocorticoid exposure has been detected in the lateral nucleus (LN) (120, 121). Contrarily, an increased number of neuronal connections and microglia was detectable upon glucocorticoid challenge in the basolateral nucleus (BLN).

In Figure 2, we depict key effects of cytokines/inflammation and glucocorticoids on microglia cells and neurons in the prefrontal cortex, hippocampus and amygdala of the fetal brain. We deliberately focused on these specific brain regions due to their specific relevance for the mental health problems that have been described in the context of maternal stress and infection (122).

Besides the effect of glucocorticoids and cytokines on neurogenesis, synaptogenesis, axon growth etc., these mediators can also exert indirect effects on these fundamental processes of fetal brain development, i.e., by altering the concentration or availability of neurotransmitters. The primary excitatory neurochemical in the central nervous system, glutamate, which acts through different types of metabotropic glutamate receptors (mGluR) has been investigated in a number of studies (123, 124, 125). Indeed, a variant of the Kozak sequence of exon 1 of the mGluR 3 could be associated with e.g., bipolar affective disorder (126). Moreover, a reduced expression of mGluR receptors in the hippocampus has been observed in response to prenatal stress (127–129). Brain functions such as mood, satiety, sleep, body temperature and nociception are also critically dependent on the serotonergic system, and prenatal challenges have been shown to interfere with the rate of serotonin synthesis (130), the number of serotonin positive cells (131) and its receptor density (132) in the offspring's brain. Further, the cholinergic system is important in regulating brain function (e.g., learning and short-term memory) (133) that has been shown to be impacted in the offspring in the context of maternal stress and infection during pregnancy. Some evidence from rodent models support the notion that prenatal stress challenges interfere with the release of cholinergic factors in the offspring, such as acetylcholine (134), whereby changes of behavior have not been evaluated in this study.

### Introducing a novel pathway in the developmental origin of neurocognitive functions and psychiatric disorders: the impact of maternal microchimeric cells

Maternal microchimeric cells, which are vertically transferred from mother to fetus during pregnancy and—at least in part—during lactation (135–137), can be detected in the offspring's brain during fetal and adult life. Hence, maternal microchimeric cells have the potential to modulate brain development and the risk for neurodevelopmental disorders. To date, no insights are available on the role of maternal microchimeric cells in brain development and their potential ability to tailor the nervous system individually. Moreover, brain structures where maternal microchimeric cells may abundantly populate have not yet been identified. However, since maternal microchimeric cells have been identified as maternal immune cells of the innate and adaptive immune response, they hold the strong potential to shape neurons by acquiring a phagocytic phenotype, akin to offspring's microglial cells. Future studies should aim at elucidating the functional role of maternal microchimeric cells on the developing brain and to understand whether they may modulate the risk for brain-related disorders.

Given the plethora of mediators that may be functionally involved in shaping brain development and subsequent function, whilst being altered upon maternal stress, infection and other prenatal conditions (138), it is not surprising that we are far from fully understanding the developmental origin of neurocognitive functions and brain disorders. Also, it seems unlikely that single mediators determine a clear-cut “good or bad” outcome. It is more likely that the mediators we here proposed act synergistically in modulating brain development and subsequent function with an advantageous or disadvantageous outcome. Hypothetically, this synergistic cross talk could involve the expression of glucocorticoid receptor on maternal microchimeric cells or the release of cytokines from maternal microchimeric cells entering the fetal brain. The longevity of such cells would surpass the short-term effect that could result from the potential transplacental transfer of cytokines itself, as cytokines are rapidly metabolized.

### Functional impact of vertically transferred maternal markers on the developing brain

In response the environmental challenges, altered levels of maternal markers that cross the placental barrier may affect the developing brain by inducing epigenetic alterations of somatic cells (139–142) Persistent epigenetic differences triggered by the prenatal exposure to stress challenges in humans include increased and decreased methylation of insulin-like growth factor 2 or the glucocorticoid receptor gene (NR3C1) in brain cells, depending on the timing of exposure (143, 144). Prenatal distress has been associated with hyper- as well as demethylation of specific regulatory sites in key genes involved in stress processing, such as the glucocorticoid receptor (144–146). Similarly, findings arising from mouse models on maternal immune activation during pregnancy include the observations of a hypoacetlyation of e.g., genes modulating neuronal development, synaptic transmission and immune signaling in the cortex region in exposed offspring (147), as well as sex-specific DNA hypomethylation in the hypothalamus of females (148), specifically affecting the promoter region of methyl CpG-binding protein 2, which is associated with neurodevelopmental disorders (149). Interestingly, prenatal immune activation in mice could be linked to hypermethylation of glutamic acid decarboxylase 1 and 2 in the brain (150), associated with altered behavior.

Strikingly, mouse models have revealed that alterations of brain function can be passed on to the next generations (151), suggesting that underlying epigenetic alterations triggered by prenatal challenges may be intergenerationally inherited. This notion could provide an explanation for the increasing incidence of behavioral disorders (152). Moreover, it implies that exposure of the mother to environmental challenges during pregnancy may not only directly interfere with fetal brain development, but also affects fetal primordial germ cells (153), which may subsequently interfere with brain development in the generation of grandchildren. Primordial germ cells undergo sequential epigenetic events, which are distinct from fetal somatic cells, hereby preserving the plasticity required for the generation of gametes (154–157). Once the offspring reaches adulthood and such oocytes are fertilized, the resulting zygote again undergoes significant epigenetic reprogramming, which includes the demethylation of the maternal and paternal genome, followed by a genome-wide *de novo* methylation (158).

Animal data indicate the possibility of transmission of behavioral traits mediated by epigenetic modifications through the maternal, as well as paternal germ line (159–162), implying the generation of oocytes and sperm may be equally affected. Intriguingly, how epigenetic changes induced by environmental challenges can be maintained throughout the multiple epigenetic reprogramming events physiologically occurring during reprogramming of primordial germ cells and the zygote is still largely elusive. Insights from mouse studies provide a first glimpse, as they reveal that certain regions of the genome, i.e., differentially methylated regions, are resistant to zygotic reprogramming (158). However, future research is required to identify pathways of intergeneration epigenetic inheritance of altered brain function in the offspring, aiming also to differentiate between *de novo* acquired epigenetic alterations from those inherited through the germ line. Besides such intergenerational inheritance of epigenetic marks, the possibility of transgenerational inheritance of epigenetic changes to one further generation of descent, the great-grand generation, has been considered. The primordial germ cells of the forth generation would not have been directly exposed to the environmental challenges or mediators released by the great-grandmother during pregnancy. However, to date, convincing evidence of transgenerational inheritance of epigenetic marks is only available from botany research using plants, whilst confirmation in mammals is somewhat elusive (163).

Besides such epigenetic pathways, the brain as target tissue for prenatal challenges may be affected in its electrical synchrony, which is defined as the coordinated oscillatory activity and neural firing rate between connected brain areas. These links are a prerequisite to execute cognitive tasks (164, 165). Interestingly, prenatal exposure to maternal inflammation or stress impairs oscillatory synchronicity (166), which commenced already during developmental stages in a mouse model of neuropsychiatric disorders (167–169) and affected spatial memory tasks (170, 171).

### Outlook

**Figure 3:**
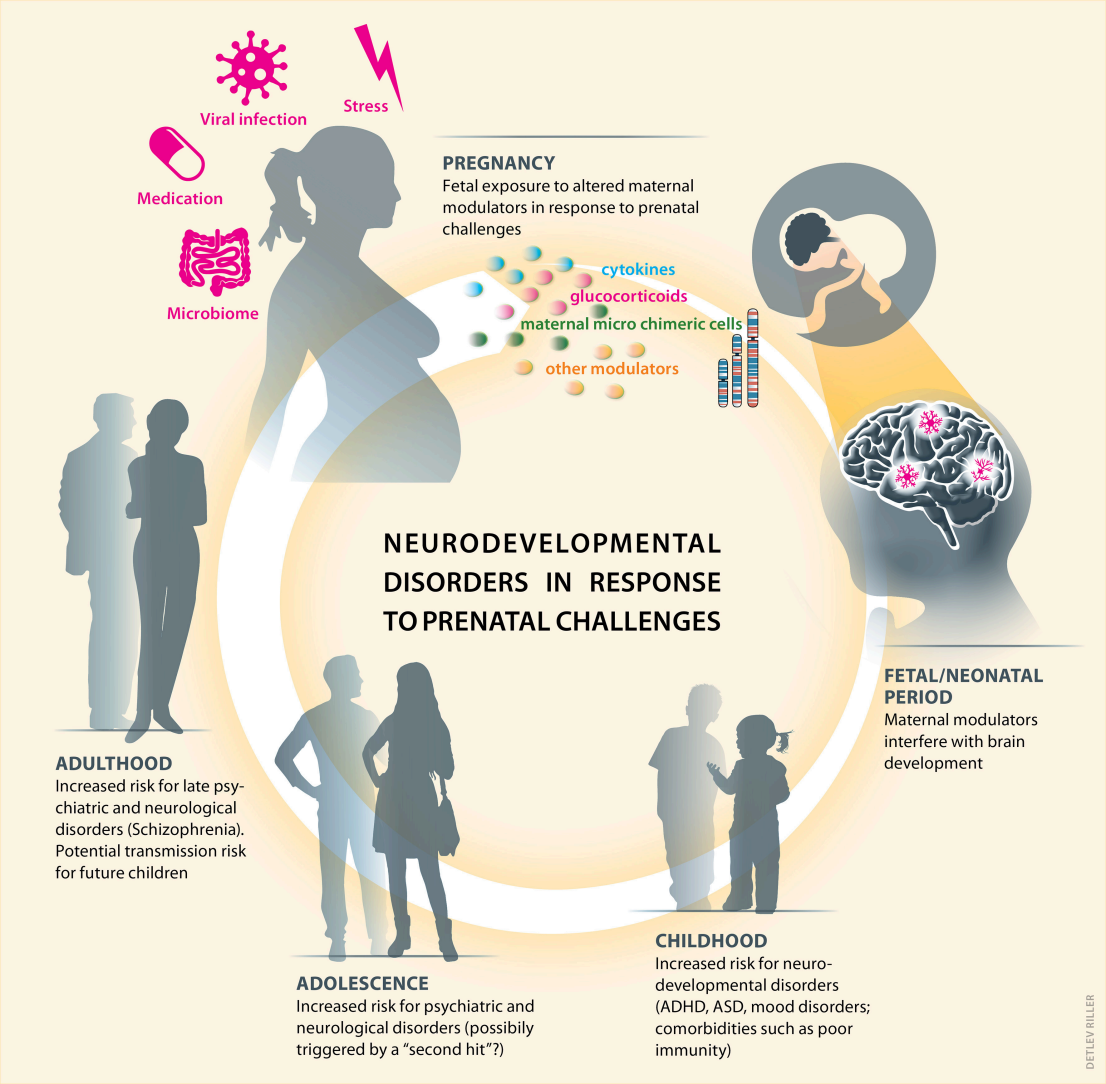
Prenatal challenges and related alterations of immune and endocrine markers can prime postnatal neurodevelopmental disorders. Maternal well-being and health can be challenged during pregnancy, e.g., by distress or infection. This subsequently leads to increased cytokines and glucocorticoids levels and potentially to altered frequencies or phenotypes of maternal microchimeric cells in the offspring. Upon entering the fetal brain, such vertically transferred maternal modulators can significantly interfere with physiologically occurring brain development. A combination of genetic susceptibility and disturbed brain development can subsequently increase the risk for neurodevelopmental disorders in childhood. Subsequent postnatal environmental challenges —drug abuse, trauma, infection, others—may perpetuate such prenatally triggered risk for neurodevelopmental disorders, psychiatric and neurological diseases during adolescence and adulthood, which can also be passed on to the next generation.

Higher cognitive functions such as planning, self-regulation, memory, learning, and emotional processes result from a complex, tailored, and precisely shaped large-scale communication of neuronal networks (172). These neuronal networks begins to develop prenatally and disturbances of such developing neural systems during pregnancy can disrupt brain development via the vertical transfer of maternal markers, such as cytokines, glucocorticoids or microchimeric cells of the maternal immune system. Subsequently, the risk for mental disorders and diseases can increase in the offspring (Figure 3). As most of the studies are correlative, future research should aim to investigate causalities between maternal factors and children's health outcome. Clearly, adverse postnatal childhood experiences can further aggravate such cognitive and behavioral dysfunctions (173–178) and thus, should be considered in experimental designs and observational studies.

## Author contributions

SS and PA developed the structure of the review article, SS provided the first draft, which was amended by CB on aspects including prenatal cytokines and epigenetic pathways, by IH-O by insights on brain development and by PA on issues related to maternal immune adaptation to pregnancy. All authors have been involved in the interpretation of published evidence, critically revised the manuscript and gave their final approval of the version to be published.

### Conflict of interest statement

The authors declare that the research was conducted in the absence of any commercial or financial relationships that could be construed as a potential conflict of interest.
